# Silk Fibroin Nanoparticle Functionalization with Arg-Gly-Asp Cyclopentapeptide Promotes Active Targeting for Tumor Site-Specific Delivery

**DOI:** 10.3390/cancers13051185

**Published:** 2021-03-09

**Authors:** Elia Bari, Massimo Serra, Mayra Paolillo, Eric Bernardi, Sara Tengattini, Filippo Piccinini, Cristina Lanni, Marzio Sorlini, Giovanni Bisbano, Enrica Calleri, Maria Luisa Torre, Sara Perteghella

**Affiliations:** 1 Department of Drug Sciences, University of Pavia, Viale Taramelli 12, I-27100 Pavia, Italy; elia.bari@unipv.it (E.B.); massimo.serra@unipv.it (M.S.); mayra.paolillo@unipv.it (M.P.); eric.bernardi01@universitadipavia.it (E.B.); sara.tengattini@unipv.it (S.T.); cristina.lanni@unipv.it (C.L.); giovanni.bisbano01@universitadipavia.it (G.B.); enrica.calleri@unipv.it (E.C.); sara.perteghella@unipv.it (S.P.); 2 IRCCS Istituto Romagnolo per lo Studio dei Tumori (IRST) “Dino Amadori”, Via Piero Maroncelli 40, I-47014 Meldola, Italy; filippo.piccinini@irst.emr.it; 3 Department of Innovative Technologies, SUPSI, Lugano University Centre, Campus Est, Via la Santa 1, 6962 Viganello, Switzerland; marzio.sorlini@supsi.ch; 4 PharmaExceed Srl, Piazza Castello 19, I-27100 Pavia, Italy

**Keywords:** silk fibroin nanoparticles, RGD, curcumin, anticancer, active targeting

## Abstract

**Simple Summary:**

Many tumor cell types overexpress integrins, a glycoprotein, on their cell membranes. The tripeptide motif Arg-Gly-Asp (RGD) is well-known for being recognized by the integrin superfamily members and can thus be used to actively target nanoparticles containing cytotoxic drugs directly to the tumor cells. According to this strategy, the antitumor activity is boosted, and healthy organs are spared. In this paper, silk fibroin, a naturally derived protein, has been used to prepare nanoparticles (SFNs) functionalized on their surface with RGD. In vitro experiments revealed that functionalization of SFNs with RGD provided active internalization by tumor cells overexpressing integrin receptors. Therefore, RGD-SFNs may be used for tumor-specific delivery of anticancer drugs.

**Abstract:**

Arg-Gly-Asp (RGD)-based cyclopentapeptides (cRGDs) have a high affinity towards integrin αvβ3 and αvβ5, which are overexpressed by many tumor cells. Here, curcumin-loaded silk fibroin nanoparticles (SFNs) have been functionalized on the surface with cRGD to provide active targeting towards tumor cells; a “click reaction” between the RGD-based cyclopentapeptide carrying an azide group and triple-bond-functionalized nanoparticles has been exploited. Both naked and functionalized SFNs were less than 200 nm in diameter and showed a round-shaped morphology but, after functionalization, SFNs increased in size and protein molecular weight. The functionalization of SFNs’ surfaces with cRGD provided active internalization by cells overexpressing integrin receptors. At the lowest concentration tested (0.01 mg/mL), functionalized SFNs showed more effective uptake with respect to the naked by tumor cells that overexpress integrin receptors (but not for non-overexpressing ones). In contrast, at higher concentrations, the non-specific cell membrane protein–particle interactions are promoted and coupled to specific and target mediated uptake. Visual observations by fluorescence microscopy suggested that SFNs bind to integrin receptors on the cell surface and are then internalized by endocytosis. Overall, SFN functionalization provided in vitro active targeting for site-specific delivery of anticancer drugs, boosting activity and sparing healthy organs.

## 1. Introduction

Cancer is one of the major disease burdens worldwide. Among the primary cancer treatment modalities, chemotherapy has been widely performed in the clinic because of its convenient and straightforward process [1] and still has a significant role in cancer treatment [2]. Regarding the pharmacotherapy of many forms of cancer, cytotoxic drugs are commonly administered systemically, thus distributing non-specifically through the entire body and causing severe side effects. In this regard, drug delivery systems (DDS) may be used for site-specific delivery of anticancer drugs, boosting activity and sparing healthy organs [3].

Among current drug delivery systems, nanoparticles have shown great potential in recent years [4,5,6]. Silk fibroin (SF) emerges among the natural polymers, specifically those characterized by a protein structure, which have been extensively investigated to produce nanoparticles. SF stands out due to its excellent biocompatibility, biodegradability, ability to self-organize, easy functionalization and biological features [7]. In this regard, our group previously prepared SF-based nanoparticles (SFNs) for the effective intercellular delivery of various drugs [8,9,10]. Once intravenously injected, SF nanoparticles mainly accumulate in the tumor tissues via passive drug targeting based on the well-known enhanced permeation and retention effect (EPR). As the basis of this effect, there is an impaired permeability of the vascular network surrounding the tumor mass; the presence of “windows” with size between 200 nm and 2 µm allows nanosystems to cross the basolateral membrane and enter into the tumor mass [11]. Therefore, this mechanism’s driving forces are the hemodynamic features of the nanoparticle and their diffusive behaviors.

Interestingly, the nanoparticle accumulation into the tumor can be increased by adopting active targeting strategies. An actively targeted nanoparticle is usually obtained by adding ligands’ surfaces for recognition by specific receptors/antigens on target cells [12]. In this regard, integrins may play a pivotal role in tumor targeting because of their overexpression on many tumor cell types and neo-angiogenic vessels [13,14]. Integrins are transmembrane glycoproteins, which consist of two subunits, α and β. They are primarily involved in cell adhesion mechanisms because of their interaction with the extracellular matrix (ECM) proteins. As a result, integrins play an essential role in both physiological and pathological conditions, and notably, disease progression in various tumor types is correlated with the overexpression of the integrins αvβ3, αvβ5, α5β1, α6β4, α4β1 and αvβ6 [15,16].

The coating of nanoparticles with integrin ligands has been explored to guide cytotoxic reagents directly to the cancer cell surface [17,18]. In this regard, the tripeptide motif Arg-Gly-Asp (RGD) is well-known for being recognized by the integrin superfamily members. Serra and colleagues recently proved that 7,5-fused azabicycloalkane systems could force an embedded RGD sequence to adopt conformations suitable for receptor binding [19,20,21]. In particular, the aforementioned 7,5-bicyclic scaffolds are useful intermediates for the synthesis of RGD-based cyclopentapeptides (cRGDs) with high affinity towards integrin αvβ3 and αvβ5 [22,23,24,25].

Given these premises, this study aims to fabricate curcumin-loaded SF nanoparticles (SFNs), conjugated with cRGDs, to achieve active tumor targeting towards various cancer cell lines. Curcumin is here selected as a drug model and for its anticancer effects [26]. In the first part of the manuscript, curcumin-loaded SFNs were prepared by a desolvation technique, functionalized with cRGD, and then characterized. Then, both naked and functionalized SFNs were tested in vitro, on different cell lines, evaluating the effect on cell viability and cellular uptake.

## 2. Materials and Methods

### 2.1. Materials

Acetone, Azide-fluor 545, collagenase IA, curcumin, dimethylsulfoxide (DMSO), glutaraldehyde, ethanol, lithium bromide, methanol, potassium chloride, potassium dihydrogen phosphate, sodium carbonate, sodium hydroxide, 3-(4,5-dimethylthiazol-2-yl)-2,5-diphenyl-tetrazolium bromide (MTT) and Millipore’s Amicon^®^ Ultra filters were purchased from Sigma-Aldrich (Milan, Italy). Dialysis cellulose tubes were purchased from Visking, Medicell Membranes Ltd. (London, UK). Moreover, 70 μm nylon meshes were purchased from Greiner Bio-One GmbH (Kremsmunster, Austria). Natriumazide and sodium sulphate were from Carlo Erba Reagenti (Milan, Italy). Caucasian colorectal adenocarcinoma cells (Caco-2), human cervix cancer cells (HeLa) and human urinary bladder (ECV) cancer cells were purchased from the European Collection of Authenticated Cell Cultures Cell Bank (ECACC, Salisbury, UK). All reagents used for cell cultures were purchased from Euroclone (Milan, Italy).

### 2.2. Silk Fibroin Extraction

SF was extracted by *Bombyx mori* cocoons according to previously reported procedures [27,28]. Briefly, after being cut into pieces of 1 × 1 cm, cocoons were degummed in 0.02 M sodium carbonate (Na_2_CO_3_) for 30 min. Then, the degummed fibers were washed in distilled water (37 °C) and dried at room temperature. A 9.3-M LiBr aqueous solution was prepared and used to dissolve SF fibers at 60 °C for 4 h. The obtained solution was placed into dialysis cellulose tubes (3–5 kDa Molecular Weight Cut-Off, MWCO) and dialyzed against distilled water at room temperature for 72 h. The SF final concentration was 8.16% *w*/*v* and was determined by freeze-drying known SF volumes (Modulyo^®^ Edwards Freeze dryer, Kingston, NY, USA) at −50 °C, 8 × 10^−1^ mbar for 72 h.

### 2.3. Silk Fibroin Nanoparticle Preparation

SF nanoparticles (SFNs) were prepared by a desolvation method according to the procedures previously reported by our group [8,9]. SF aqueous solution was diluted to 1.5% *w*/*v* and then added dropwise to the acetone, where curcumin was previously solubilized at a concentration of 0.08 mg/mL. SFN suspension was dialyzed against distilled water at room temperature for 72 h using dialysis cellulose tubes (12 kDa MWCO), freeze-dried at 8 × 10^−1^ mbar and −50 °C and stored at room temperature until use (maximum 2 months).

### 2.4. Functionalization of Silk Fibroin Nanoparticles with Alkyne Linker 4 and Bioconjugation with Azide-Fluor 545

**Figure 1:**
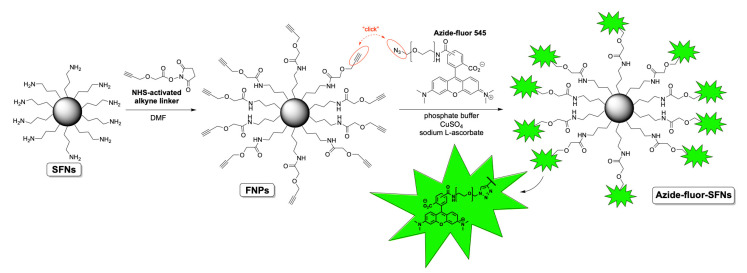
Scheme of SFNs’ bioconjugation with Azide-fluor 545.

Before functionalizing SFNs with RDG, we performed bioconjugation with a fluorescent compound named Azide-fluor 545 (Molecular Weight 631.70 g/mol) (Figure 1). Azide-fluor 545, a modified rhodamine derivative, was selected as a fluorescent probe to ascertain the effective binding of the alkyne linker to the nanoparticles’ surfaces and evaluate the percentage of functionalization. Moreover, as the Azide-fluor’s molecular mass and the length of the linker bearing the azide function are very similar to that of cRGD-azide derivative 3, we thought it could be a suitable study compound to verify the applicability of our conjugation protocol. Fibroin nanoparticles (20 mg, Lys: 80 nmol/mg) were suspended in phosphate buffer (1 mL, pH = 7.4) for 1 h to ensure their complete hydration. A 10-mg/mL solution of NHS-activated linker 4 was then added (50 μL, 0.0017 mmol). After 24 h, the alkyne-functionalized nanoparticles were purified through dialysis against water at room temperature for 48 h. The alkyne-functionalized nanoparticles (20 mg, Lys: 80 nmol/mg) were suspended in phosphate buffer (1 mL, pH 7.4) for 1 h to ensure their complete hydration. Azide-fluor 545 (1.4 mg, 0.0024 mmol), a 12 mg/mL solution of CuSO4 (100 μL, 0.0000481 mmol) and 52-mg/mL solution of sodium l-ascorbate (100 μL, 0.00262 mmol) were added. After 72 h, the Azide-fluor nanoparticles were purified through dialysis against distilled water at room temperature for 48 h.

### 2.5. Determination of Azide-Fluor 545 Conjugated on SFNs

The quantification of Azide-fluor conjugated on SFNs was evaluated with spectrophotometric analysis (Synergy HT, BioTek, Swindon, United Kingdom) at 554 nm. First, 2 mg of SFNs was suspended in 300 µL of distilled water, maintaining magnetic stirring overnight. After incubation, nanoparticles were centrifuged at 3250× *g* for 10 min, and supernatants were analyzed. A calibration curve (correlation coefficient R^2^ > 0.9899) was obtained to measure the Azide-fluor concentration; we consider the Azide-fluor concentration range of 0–10 µg/mL (deionized water was considered as control solution). Each measurement was performed in triplicate.

### 2.6. Silk Fibroin Nanoparticle Functionalization with RGD

**Figure 2:**
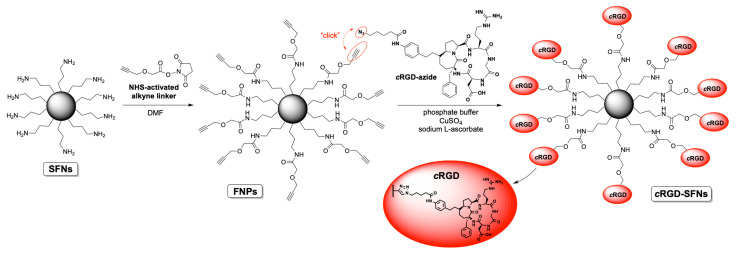
Scheme of SFNs functionalization with RGD.

The synthesis of cRGD-functionalized SFNs (cRGD-SFNs) was carried out by exploiting a “click reaction” between the azide group of the RGD-based cyclopentapeptide and a triple bond on the surface of SFNs (Figure 2). SFN functionalization was performed using the cRGD-azide derivative 3, synthesized as described in Text S1. Triple-bond-functionalized nanoparticles (FNPs) were prepared by performing a condensation reaction between an NHS-activated linker bearing a terminal alkyne moiety and SFNs (for details, see Text S1). Lysine residue on the fibroin’s surface, 80 nmol/mg of protein on average [17], was chosen as the linker’s anchoring point through an amide bond formation. The obtained FNPs were then subjected to dialysis to remove all non-covalently linked small molecules (cut-off 3–5 kDa MWCO). Similarly, the azide moiety was easily installed on the cRGD derivative by subjecting the side chain’s amine group to a condensation reaction with an NHS-activated linker endowed with a terminal N_3_ group. The key step, i.e., the Huisgen cycloaddition between FNPs and the azide-functionalized cRGD, was carried out in phosphate buffer in the presence of CuSO_4_ and sodium l-ascorbate. The final dialysis (cut-off 3–5 kDa MWCO) of the reaction mixture allowed the obtainment of pure cRGD-SFNs.

### 2.7. Characterization of Nanoparticles

Both nude and functionalized SFNs were characterized as follows.

#### 2.7.1. Production Yield, Drug Loading and Encapsulation Efficiency Evaluation

SFN production yield (Y%) was calculated as follows:

Y% = (total weight nanoparticles)/(weight of polymer + weight of drug) × 100

The amount of curcumin loaded into SFNs was quantified by spectrophotometric analysis. Briefly, SFNs were dissolved in 96% *v*/*v* ethanol (0.1 mg/mL), maintaining mild magnetic stirring in the dark, and the absorbance was measured at 254 nm by a spectrometer (Uvikon 860, Kontron Instruments, Zurich, Switzerland). Ethanol was considered as blank. To extrapolate the drug content, a calibration curve was prepared using curcumin in the concentration range 0.25–10.00 μg/mL; R^2^ was 0.9957. The drug loading (% *w*/*w*) was calculated, dividing the total drug content (extrapolated from the calibration curve) and the concentration of analyzed SFNs. All experiments were performed in triplicate.

The encapsulation efficiency (EE%) was calculated as the percentage ratio between the drug entrapped in SFNs and the drug dissolved in the acetone solution.

#### 2.7.2. Nanoparticle Size Distribution and Zeta Potential

Particle size and size distribution of both naked and functionalized SFNs were measured by Nanoparticle Tracking Analysis (NTA, NanoSight NS300, Malvern Panalytical, Grovewood Rd, WR14 1XZ, Great Malvern, Worcestershire, United Kingdom). The instrument was fitted with a flow-cell top plate and a 405 nm laser. The temperature was set at 25 °C and the detection angle at 90°. Before carrying out NTA analyses, SFNs were dispersed in water, vortexed and sonicated for 15 min. Measurements were repeated 3 times for each sample, and results were analyzed with the NTA software 3.0.

The polydispersity index (PDI) and zeta potential of both naked and functionalized SFNs were measured using ZetasizerNano ZS (Malvern Instruments 15 Ltd., Worcestershire, United Kingdom). Aqueous suspensions (1 mg/mL) of SFNs were prepared and allowed to equilibrate overnight at room temperature with gentle stirring. Then, the nanoparticle dispersions were filtered with a 0.45 μm filter and analyzed. The measurements were performed in triplicate for each sample. To define the Z-potential of the nanoparticles, 1 mM KCl was added to the aqueous suspension of 1 mg/mL.

#### 2.7.3. Morphological Evaluation by Scanning Electron Microscopy (SEM)

Morphology of both naked and functionalized SFNs was evaluated using SEM (MIRA3, Tescan, Brno, Czech Republic). Before the analysis, freeze-dried samples were gold-sputter-coated under argon.

#### 2.7.4. SEC-UV Analysis

A 9.3-M LiBr aqueous solution was prepared and used to solubilize both naked and functionalized SFNs (2 mg/mL) at 60 °C for 4 h. The solution obtained was ultrafiltered 2 times (10 min each) at 10,000× *g* and 4 °C using Millipore’s Amicon^®^ Ultra Filters (Oakville, ON, Canada) with a Nominal Molecular Weight Limit (NMWL) of 10 kDa and load capacity of 500 µL. Deionized water was used as a washing solution. The collected samples were injected into the HPLC system. The instrument was an Agilent HPLC series 1100 system (Santa Clara, CA, USA), equipped with a quaternary pump, an autosampler, a mobile phase online degasser, a thermostated column compartment and a diode array detector. The analytical column was a TSKgel SuperSW3000 (4.6 × 300 mm). SEC analysis was performed according to a previously reported method [29]. The mobile phase was composed of 0.1 M Na_2_SO_4_ and 0.05% (*w*/*v*) NaN_3_ in 0.1 M phosphate buffer, pH 6.7. The elution was performed in isocratic conditions at the constant flow of 350 μL/min. The column temperature was maintained at 25 °C, and the injection volume was set at 5 μL. UV absorption was monitored at 280 nm. Calibration was performed using protein standards (see Table S1 and Figure S1). Analyses were performed in triplicate.

### 2.8. In Vitro Biological Assays

#### 2.8.1. Cell Culture

Mesenchymal stem cells (AD-MSCs) were isolated from adipose tissue samples collected from patients undergoing abdominoplasty surgery after informed consent was obtained (ASST Grande Ospedale Metropolitano Niguarda, Milan, Italy) and according to previously reported procedures [30,31,32]. AD-MSCs were expanded in monolayer conditions in Dulbecco’s modified Eagle medium (DMEM) F12, 10% (*v*/*v*) FBS, 1% (*v*/*v*) penicillin/streptomycin and 1% (*v*/*v*) amphotericin B. MSCs fulfilled adherence to the International Society for Cellular Therapy criteria [33].

Human fibroblasts (HF) were obtained from informed donors subjected to skin biopsy. Skin samples were digested as previously reported [34]. Obtained fibroblasts were cultured and expanded in DMEM High-Glucose (DMEM-HG) supplemented with 10% FBS, antibiotics and L-glutamine (1.8 mM).

Caco-2 cells were cultured at 37 °C and 5% CO_2_ in DMEM-HG supplemented with 10% fetal bovine serum, non-essential amino acids (NEAA) 100×, penicillin 100 U/mL, streptomycin 100 μg/mL, amphotericin 0.25 μg/mL, glutamine 4 mM, sodium pyruvate 1 mM.

HeLa were cultured in DMEM with a low content of glucose (1 g/L), supplemented with 10% (*v*/*v*) FBS, 1 mM glutamax, 1% *v*/*v* antibiotics (penicillin-streptomycin) and 1 mM pyruvate at 37 °C, under a humidified atmosphere containing 5% CO_2_.

ECV cells were maintained in complete DMEM medium containing 10% FBS in a humidified 5% CO_2_ atmosphere at 37 °C.

#### 2.8.2. Real-Time qRT-PCR

To assess whether MSCs, HF, Caco-2, HeLa and ECV cells expressed integrin subunits, the mRNA expression of αv, α5, β1, β3, β5 was evaluated as previously described [25]. In brief, RNA was extracted from the cell lines by Qiazol (Qiagen, Milan, Italy), followed by a DNAse digestion step. The primers were designed using the Primer3 Input software (version 4.1.0). The specificity of each primer was checked by BLAST analysis. Table 1 reports the primers used for integrin subunits in quantitative real-time RT-PCR reactions [25]. At the end of the reaction, a melting curve analysis was carried out to check for primer dimers’ presence. GAPDH was used as a housekeeping gene. Experiments were performed on three different cell preparations, and each run was analyzed in duplicate. Data are expressed as fold change by the ∆∆-Cq method, using normal human fibroblasts as a reference and GAPDH as housekeeping gene, as described in [25].

#### 2.8.3. Cell Metabolic Activity Evaluation

The cytocompatibility of both naked and functionalized SFNs was evaluated by the MTT test on all the cell lines reported in Section 2.8.1. Briefly, cells were seeded in a 96-well plate (5000 cells/cm^2^) and cultured with their culture media as described in Section 2.8.1. After 24 h, the supernatants were replaced with 100 µL of culture medium (not supplemented with FBS) containing SFNs at the final concentrations of 0.4, 0.2, 0.05 and 0.01 mg/mL. After 24 h of incubation, supernatants were discarded, cells were then washed with PBS, and 100 µL of MTT solution (0.5 mg/mL) was added to each well. After 3 h of incubation, the MTT solution was removed, and 100 µL of DMSO was added. Cells not treated with SFNs were considered as controls (100% of metabolic activity). The absorbance was measured by a microplate reader (Synergy HT, BioTek, Swindon, United Kingdom) at 570 and 670 nm (reference wavelength). Each condition was tested in triplicate, and the percentage of cell metabolic activity was calculated as follows:

Cell metabolic activity (%) = 100 × (Abs_sample_/Abs_ctr_)

where Abs_sample_ is the mean value of the measured absorbance of the tested samples, and Abs_ctr_ is the mean value of the measured absorbance of cells not incubated with SFNs. All experiments were performed in triplicate.

#### 2.8.4. Cell Uptake Evaluation

Cellular uptake was evaluated, exploiting the auto-fluorescence property of curcumin [8]. Cells were seeded in 96-well plates at a density of 5000 cells/cm^2^. After 24 h of incubation with culture medium (as reported in Section 2.8.1) at 37 °C, 5% CO_2_, SFNs were added to each well (at the concentration reported in Section 2.8.3). At the established times (30, 60, 120, 180 or 360 min), cells were washed with PBS, and fluorescence intensity was measured using a microplate reader (Synergy HT, BioTek, UK) at 485 nm excitation and 528 nm emission. Cells not treated with SFNs were considered as controls. All experiments were performed in triplicate.

The experiments were repeated on Caco-2 and ECV cells at 60, 180 and 360 min. These two cell lines display opposite integrin subunit expression patterns: Caco-2 cells express very low integrin subunit mRNA levels, particularly β3, while in ECV, integrin mRNA levels appear to be higher, and β3 is very abundant (see Table 2). Therefore, these two cell lines represent a suitable model to investigate RGD nanoparticle uptake. Then, the cells grown were fixed using a 3% (*v*/*v*) glutaraldehyde solution in PBS for 3 h at 4 °C and washed four times with PBS. Naked and functionalized nanoparticles taken up by fibroblasts ECV and Caco-2 cells were visualized under a Zeiss Axioskop 40 microscope (Carl Zeiss, Jena, Germany) equipped with a 40 × 0.75 objective lens and an RT Slider camera (Diagnostic Instruments, Sterling Heights, MI, USA).

#### 2.8.5. Fluorescence Microscopy Image Analysis

The microscopy fluorescence images acquired were firstly pre-processed using ImageJ [35] and then analyzed with MatLab (The MathWorks, Inc., Natick, MA, USA). Precisely, (I) all the images were vignetting corrected using CIDRE [36]. Then, (II) the foreground (i.e., intensity values referring to the cells) was segmented from the black background by applying (a) a global threshold determined computing the peak of the cumulative histogram of the images and (b) some morphological operators to smooth the foreground binary masks obtained. Finally, (III) the image-based average intensity values were extracted using a MatLab script provided with the ImageJ macro designed to compute the foreground binary masks and the table reporting the intensity values (see Table S2). The original 8-bit microscopy fluorescence images, the ImageJ macro, the MatLab script and the foreground binary masks obtained are provided as a FigShare collection named “2021_BariElia_Collection1”, publicly available at [37].

### 2.9. Statistical Analysis

Raw data were processed by STATGRAPHICS XVII (Statpoint Technologies, Inc., Warrenton, VA, USA). For data with a normal distribution, a linear generalized analysis of variance model (ANOVA) was used and combined with Fisher’s least significant difference (LSD) procedure to evaluate the differences between the groups. For all the analyses, the statistical significance was set at *p* < 0.05. Unless otherwise specified, data are expressed as mean ± standard deviation.

## 3. Results and Discussion

SFNs were prepared by a desolvation method. The addition of acetone as a desolvating agent reduces fibroin chains’ hydration until fibroin protein precipitates in the form of nanoparticles [7]. After freeze-drying, the process yield (%) in the preparation of SFNs was 70.8%, and the curcumin drug loading was 1.25 ± 0.00584% (*w*/*w*), with an encapsulation efficiency of 83.58%. These results agree with our previous works [9,38], thus indicating the reproducibility of the preparation method. SFNs were then functionalized with cRGD, exploiting a “click reaction” between the RGD-based cyclopentapeptide carrying an azide group and triple-bond-functionalized nanoparticles. At first, the feasibility of the reaction was demonstrated. To this end, naked SFNs were conjugated with Azide-fluor 545, a fluorescent compound selected to demonstrate both the biding of alkyne linker to SFNs and the functionalization percentage. The obtained results confirmed the effective binding and a functionalization of 0.23% *w*/*w*. The alkyne linker, consequently the Azide-fluor, selectively reacted and attached the Lysine residues on SFNs’ surfaces with a 1:1 stoichiometric ratio reaction. Starting from this assumption, we considered the molecular weight of Azide-fluor (631.70 g/mol) to obtain the bioconjugation data expressed as 3.64 nmol of reacted Lysine for mg of SFNs.

**Figure 3:**
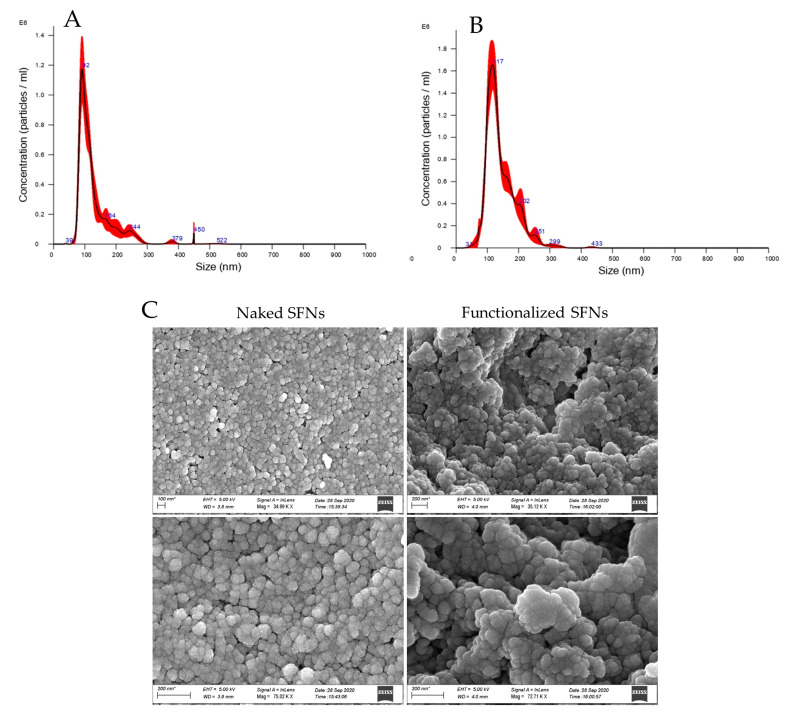
Particle size distribution for naked (**A**) and functionalized (**B**) SFNs, and morphological investigation by SEM (**C**). Scale Bar:100 or 200 nm.

After cRGD functionalization, SFN particle size was significantly increased (*p* < 0.05). Naked SFNs showed a mean diameter of 128.9 ± 2.4 nm, and the d_10_, d_50_ and d_90_ were 84.9 ± 0.8, 109.8 ± 0.9 and 201.5 ± 12.6 nm, respectively (mean values ± standard deviation, *n* = 3). For cRGD-functionalized SFNs, the mean diameter was 142.7 ± 2.7 and the d_10_, d_50_ and d_90_ were 96.7 ± 1.8, 129.1 ± 2.8 and 205.0 ± 3.3 nm, respectively (mean values ± standard deviation, *n* = 3) (Figure 3A,B). Particle size results were also confirmed by Dynamic Light Scattering (DLS) analysis. For both naked and functionalized SFNs, the PDI was approximately 0.2, and the Z-potential decreased from −17.97 to −23.7 mV after functionalization. SEM morphological investigation showed a spherical shape for both naked and functionalized SFNs (Figure 3C). Larger size was also observed by SEM for functionalized SFNs. It has to be noted that in the SEM images, functionalized SFNs showed a greater tendency to aggregate. However, this appeared to be a transitory phenomenon probably related to the removal of the solvation shell from SFNs [39]. Indeed, after suspension in water and sonication, no aggregation was observed, either for naked or functionalized SFNs.

**Figure 4:**
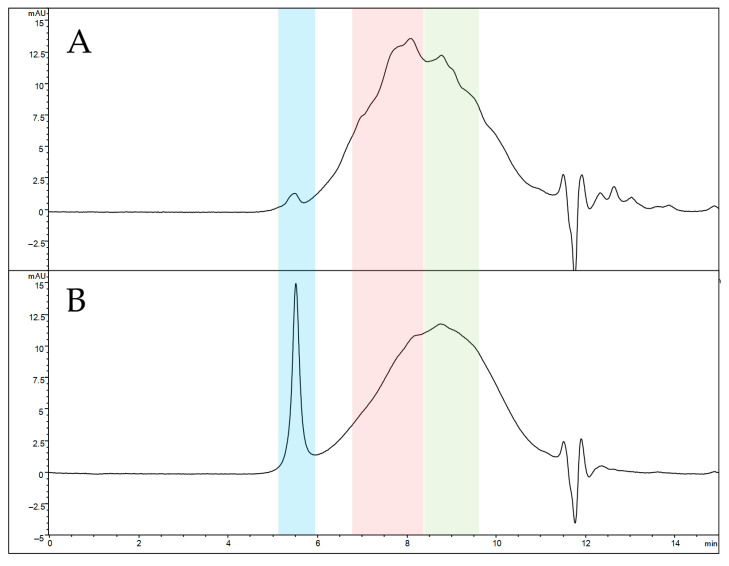
SEC-UV profiles of solubilized naked (**A**) and functionalized (**B**) SFNs. Elution windows of the three main protein populations are colored in blue (apex at 5.5 min, estimated MW greater than 700 kDa), red (apex at 8.1 min, estimated average MW of 110 kDa) and green (apex at 8.8 min, estimated average MW of 66 kDa).

Analytical size exclusion chromatography (SEC) was selected to compare the elution profiles of naked and functionalized SFNs due to its ability to elute analytes in order of their molecular size. The SEC profile of solubilized naked SFNs (Figure 4A) reveals a highly broad peak, indicating an extremely heterogeneous protein mixture. Three main protein populations seem to be present, eluting at 5.5 min (blue box), 8.1 min (red box) and 8.8 min (green box). According to the calibration curve, the estimated average MW is 66 kDa for the last eluting peak (8.8 min) and 110 kDa for the peak at 8.1 min. The first eluted peak (5.5 min) retention time falls outside the calibration range, suggesting an MW greater than 700 kDa, these species not being retained by the stationary phase. After functionalization, a significant change in the SEC profile is observed in relative abundance (Figure 4B). The peak at 8.1 min decreases in height, while the non-retained peak increases its abundance, suggesting that the functionalization induces the formation of a high-MW protein population. Overall, the increased size of nanoparticles after functionalization and the greater MW suggest proper functionalization of SFNs.

Drug release studies have been performed on both naked and functionalized SFNs, as reported in the Supplementary Material. Overall, drug release profiles were superimposable to the ones reported in our previous papers [9,38]. No significant differences in drug release profiles were observed after functionalization with cRGD (*p* > 0.05, see Text S2 and Figure S2).

Before starting treatments with naked and functionalized nanoparticles, the cells used in this study were tested for RGD-binding integrin mRNA expression by real-time qRT-PCR, as described in the Section 2; αv, α5, β1, β3, β5 integrin subunit mRNA was detected in all the cells but overexpressed by ECV and MSCs. Data reported in Table 2 were obtained by the ∆∆-Cq method [25] and report the relative integrin subunit expression of each cell line compared to normal fibroblasts. Data are expressed as fold change.

**Figure 5:**
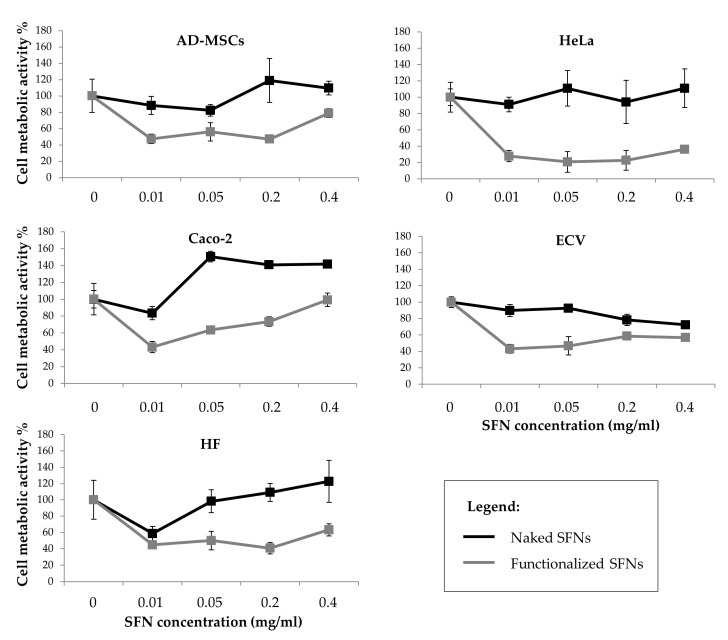
Cell metabolic activity of all considered cell lines treated with increasing doses of curcumin-loaded naked (black line) and functionalized (grey line) SFNs. Data are reported as mean values and least significant difference (LSD) intervals, ANOVA. The overlap of two LSD intervals graphically indicates the absence of significant differences (*p* > 0.05).

Figure 5 reports the percentage of cell metabolic activity values after treatment with naked and functionalized SFNs. Cells incubated with naked SFNs remained almost 100% viable relative to controls (SFN concentration = 0) for all the doses tested. SFNs functionalized with cRGD showed a more cytotoxic effect on all the cell lines (*p* < 0.05), and especially on the Caco-2 cell line, where the cell metabolic activity was reduced to 27.9 ± 6.594 already at the lowest concentration considered (0.01 mg/mL). No dose-dependent effect was observed (*p* = 0.06). It is not clear what caused the apparent decrease in the viability for functionalized nanoparticles at concentrations above 0.01 mg/mL, but since there is no dose-dependent continuous decrease, it is hard to say that the nanoparticles caused severe cytotoxicity [40].

**Figure 6:**
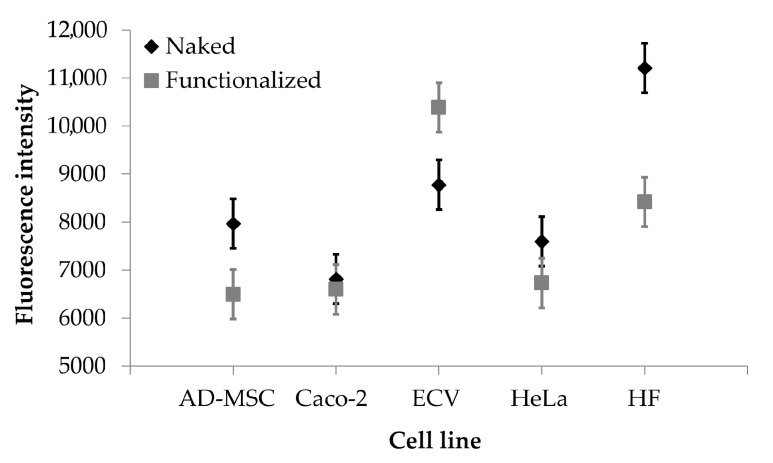
Cellular uptake results. Fluorescence intensity obtained after treatment with naked (black rhombuses) and functionalized (grey rectangles) SFNs (concentration 0.01 mg/mL). Data are reported as mean values and least significant difference (LSD) intervals, ANOVA. The overlap of two LSD intervals graphically indicates the absence of significant differences (*p* > 0.05).

The auto-fluorescence of curcumin has been exploited for quantitative analysis of nanoparticle uptake by different cell lines, overexpressing or not overexpressing the integrin receptors. Cells were incubated with naked or functionalized SFNs, and fluorescence intensity of adherent cells was evaluated as uptake index. The uptake of nanoparticles was dose-dependent for all the times considered (*p* < 0.001); at the high concentrations tested (0.05, 0.2 and 0.4 mg/mL), no significant differences were observed among the uptake of functionalized or naked SFNs by all the cell lines considered. However, at the lowest concentration tested (0.01 mg/mL), functionalized SFNs were more effectively taken up by ECV cells, which overexpress integrin receptors (Figure 6). It is likely that at higher concentrations, the nanoparticle uptake by a non-specific phenomenon is promoted and coupled to the specific and target mediated uptake. In this regard, Moore and colleagues demonstrated that the cells’ particle uptake is proportional to the concentration right outside the cells. Moreover, the concentration gradient in solution increases cell membrane protein–particle interaction, and this trend appears to be a non-specific phenomenon [41]. Therefore, by increasing the concentration, the non-specific cell membrane protein–particle interactions are increased at the expense of specific and target mediated uptake.

**Figure 7:**
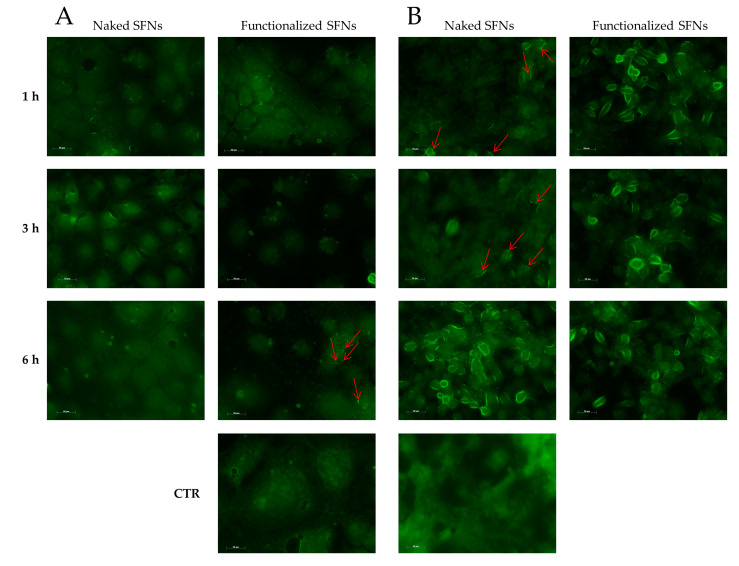
Cellular uptake of naked and functionalized SFNs by Caco-2 (**A**) and ECV (**B**) cells after incubation for 1, 3 and 6 h at 0.01 mg/mL. Red arrows indicate marked fluorescence in the proximity of cell membranes. Scale bar = 50 μm.

Cellular uptake of SFNs (at 0.01 mg/mL) was further confirmed by microscopy (exploiting the fluorescence of fibroin and curcumin) using Caco-2 (which do not overexpress integrin receptors) and ECV (which overexpress integrin receptors). For Caco-2 cells, no different behavior was revealed among naked and functionalized SFNs at 1 and 3 h. Only after 6 h of incubation, a slight internalization of functionalized SFNs is detectable as an increase in fluorescence in the vicinity of the cell membrane (Figure 7A, red arrows). For naked SFNs, an effective uptake by ECV was revealed only after 6 h of incubation; after 1 and 3 h, only slight fluorescence was detectable on the cell surface (Figure 7B, red arrows). A marked uptake was instead revealed for functionalized SFNs already after 1 h of incubation. Specifically, at the initial time points, SFNs were present mainly on the cell surface; then, after 3 h, the fluorescence became less marked in the vicinity of the cell membrane and increased towards cell cytosol. Although the actual mechanism of internalization of functionalized SFNs has yet to be determined, these observations suggest that they bind to integrin receptors on the cell surface, being thus adsorbed on the cell membrane, and are then internalized by endocytosis. This reflects a general internalization process, as reported in the literature for other nanoparticles [42,43].

**Figure 8:**
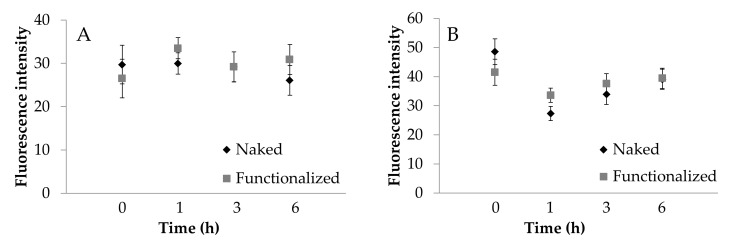
Cellular uptake of naked (black) and functionalized (grey) SFNs by Caco-2 (**A**) and ECV (**B**) cells after incubation for 1, 3 and 6 h at 0.01 mg/mL. Fluorescence intensity was extracted from the images collected by a Zeiss Axioskop 40 microscope. Data are reported as mean values and least significant difference (LSD) intervals, ANOVA. The overlap of two LSD intervals graphically indicates the absence of significant differences (*p* > 0.05).

To further confirm what was observed, the images acquired were processed to extract the average intensity values of fluorescence. ECV cells fluoresce significantly more than Caco-2 cells (Figure 8, *p* < 0.001). For Caco-2 cells, fluorescence intensity was not significantly influenced by the functionalization (Figure 8A). For ECV, a significant increase in functionalized SNP uptake was observed only after 1 h of treatment (Figure 8B). For Caco-2 cells, the treatment time was not significant (*p* > 0.05); for ECV cells, the treatment time was significant (*p* = 0.0495), and an increasing trend was detectable. These results confirm what was observed visually and support the supposed uptake mechanism.

## 4. Conclusions

Curcumin-loaded SFNs were prepared by a desolvation method and then functionalized with cRGD to provide active targeting towards tumor cells that overexpress integrin receptors. A “click reaction” was exploited between the RGD-based cyclopentapeptide carrying an azide group and triple-bond-functionalized nanoparticles. SFNs showed round-shaped morphology, and the mean diameter was 128.9 ± 2.4 and 142.7 ± 2.7 for naked and functionalized SFNs, respectively. After functionalization, a higher-MW protein population was observed by comparing the SEC elution profiles, suggesting proper functionalization. The functionalization of SFNs’ surfaces with cRGD provided active internalization by cells overexpressing integrin receptors, such as ECV. In detail, a marked uptake for functionalized SFNs was observed for ECV cells already after 1 h. In contrast, for Caco-2 cells, which do not overexpress integrin receptors, no differences were revealed between naked and functionalized SFN uptake at 1 and 3 h. Only after 6 h of incubation, a slight internalization of functionalized SFNs is detectable as an increase in fluorescence in the vicinity of the cell membrane. Overall, SFN functionalization provided in vitro active targeting for site-specific delivery of anticancer drugs.

## Figures and Tables

**Table 1:** Primers used to amplify RGD-binding integrin mRNA in qRT real-time PCR experiments.

Gene	Accession Number	Primer Sequence
v	NM_002210	F: actggcttaagagagggctgtg
		R: tgccttacaaaaatcgctga
3	NM_000212	R: tcctcaggaaaggtccaatg
		R: tcctcaggaaaggtccaatg
5	NM_002213	F: agcctatctccacgcacac
		R: cctcggagaaggaaacatca
5	NM_002205	F: cctgctgtccaccatgtcta
		R: ttaatggggtgattggtggt
1	NM_133376	F: tccaatggcttaatttgtgg
		R: cgttgctggcttcacaagta
GAPDH	NM_002046.5	R: cagcaagagcacaagaggaag
		F: caactgtgaggaggggagatt

**Table 2:** Integrin subunit mRNA expression (fold change).

Integrin Subunit	ECV	MSCs	Caco-2	HeLa
αv	1.27	1.65	0.13	0.2
α5	0.4	2.55	0.23	0.83
β1	2.85	4.03	0.34	0.28
β3	15.89	3.07	0.015	0.014
β5	0.47	2.57	0.07	0.39

Data obtained by quantitative real-time RT-PCR are expressed as fold change by the ∆∆-Cq method (2^∆∆-Cq^), using normal human fibroblasts as a reference and GAPDH as housekeeping gene. Values below 1 indicate a lower expression compared to reference cells.

## Data Availability

The original 8-bit microscopy fluorescence images, the ImageJ macro, the MatLab script and the foreground binary masks obtained are provided as a FigShare collection named “2021_BariElia_Collection1”, publicly available at: https://doi.org/10.6084/m9.figshare.c.5270543.

## Supplementary Materials

The following are available online at https://www.mdpi.com/2072-6694/13/5/1185/s1, Text S1: Synthesis and characterization of cRGD, Text S2: Drug Release Studies, Figure S1: Calibration curve derived from the analysis in SEC-UV of five protein standards, Figure S2: In vitro drug release profiles of naked and functionalized SFNs, Table S1: Data obtained from the SEC-UV analysis of five protein standards, Table S2: Values of fluorescence intensity.
