# Supplementary material for: Silk Fibroin Nanoparticle Functionalization with Arg-Gly-Asp Cyclopentapeptide Promotes Active Targeting for Tumor Site-Specific Delivery
Source: Cancers (Basel). 2021 Mar 9;13(5):1185. doi: 10.3390/cancers13051185 (PMC7967211; doi:10.3390/cancers13051185)
Supplement: Supplementary file 1 [file cancers-13-01185-s001.zip › Supplementary.docx]

Supplementary Material: Silk Fibroin Nanoparticle Functionalization with Arg-Gly-Asp Cyclopentapeptide Promotes Active Targeting for Tumor Site-Specific Delivery

Elia Bari, Massimo Serra, Mayra Paolillo, Eric Bernardi, Sara Tengattini, Filippo Piccinini, Cristina Lanni, Marzio Sorlini, Giovanni Bisbano, Enrica Calleri, Sara Perteghella and Maria Luisa Torre

Text S1. Synthesis and Characterization of cRGD

1.1. General Remarks

All chemicals were of reagent grade and were used without further purification. Reactions requiring anhydrous conditions were performed under N_2_. Yields were calculated for compounds purified by HPLC chromatography and judged homogeneous by thin-layer chromatography, NMR, and mass spectrometry.

HPLC purifications were carried out on an Agilent Technologies 1260 Infinity preparative HPLC provided with a diode array UV-vis detector, using a Waters XSelect CSH Phenyl-Hexyl column (5 µm, 150 × 30 mm) at 30 mL/min flow rate.

The HRMS spectra were acquired on a Thermo Finnigan Q Exactive instrument with API-HESI source. Samples were introduced as 0.1 mg/L solutions in MS grade methanol with a 5 µL/min flow and the following source parameters: sheath gas flow rate: 5 u.a.; aux gas flow rate: 3 u.a.; sweep gas flow rate: 0 u.a.; spray voltage: 3,50 kV; capillary temperature: 250 °C; s-lens RF level: 60.0 V; aux gas heater temperature: 0 °C.

Nuclear magnetic resonance spectra were acquired using a Bruker Avance 400 MHz spectrometer equipped with Bruker’s TopSpin 1.3 software package. The abbreviations s, d, t, q, br s, and m stand for the resonance multiplicities singlet, doublet, triplet, quartet, broad singlet, and multiplet, respectively. In the peak listing of ^13^C spectra abbreviations s and t refer to zero and two protons attached to the carbons, as determined by DEPT-135 experiments. Sample temperatures were controlled with the variable-temperature unit of the instrument. Sample temperatures were controlled with the variable-temperature unit of the instrument.

1.2. Synthesis of cRGD-Azide Derivative 3

The cRGD derivative **1** (10 mg, 0.011 mmol) was dissolved in DMF (0.2 mL, 0.05 M) at 0 °C, under nitrogen atmosphere, and the NHS-activated azide linker **2** (5 mg, 0.02 mmol) was added at the reaction mixture. The pH was adjusted to 8.0 with Et_3_N, and after 30 min, the reaction was heated at 25 °C. After 24 h the solvent was removed at reduced pressure, and the crude product was purified by HPLC (isocratic elution for 2 min with 95% water + 0.1 % CF_3_COOH then increasing gradient to 60% of acetonitrile over 20 min). The purified product was then freeze-dried to give the trifluoroacetate salt of the desired pure *c*RGD-azide derivative **3** as a white foam (10 mg, 0.010 mmol, 98% yield).

Compound 1: ^1^H NMR (D_2_O, 60 °C, 400 MHz) δ 1.68–1.79 (m, 3H), 1.81–2.32 (m, 9H), 2.42 (m, 1H), 2.52 (m, 1H), 2.62–2.78 (m, 2H)2.91 (m, 1H), 3.11 (dd, *J* = 6.7, 17.1 Hz, 1H), 3.17 (m, 1H), 3.25 (dd, *J* = 6.8, 17.0 Hz, 1H), 3.30–3.45 (m, 2H), 3.52 (d, *J* = 13.4 Hz, 1H), 3.80 (d, *J* = 13.4 Hz, 1H), 3.88 (d, *J* = 14.7 Hz, 1H), 4.29 (d, *J* = 14.6 Hz, 1H), 4.41 (dd, *J* = 8.1, 10.1 Hz, 1H), 4.65 (dd, *J* = 4.7, 8.0 Hz, 1H), 5.16 (t, *J* = 6.5 Hz, 1H), 7.34–7.40 (m, 2H), 7.56–7.77 (m, 7H); HRMS (ESI) calculated for C_37_H_50_N_9_O_7_ [M+H]^+^ 732.38277 found 732.38379 (Δ = 1.4 ppm)

Compound 3: ^1^H NMR (CD_3_OD, 400 MHz) δ 1.43–1.52 (m, 3H), 1.61–1.86 (m, 13H), 2.10–2.22 (m, 2H), 2.41 (t, 2H), 2.46 (m, 1H), 2.66–2.72 (m, 3H), 2.78 (m, 1H), 2.95 (dd, *J* = 7.2, 16.8 Hz, 1H), 3.08 (m, 1H), 3.14 (m, 1H), 3.35–3.38 (m, 3H), 3.44 (d, *J* = 13.1 Hz, 1H), 3.57 (d, *J* = 13.1 Hz, 1H), 3.96–4.12 (m, 2H), 4.33 (m, 1H), 4.92 (t, *J* = 6.6 Hz, 1H), 7.05–7.13 (m, 2H), 7.17 (d, *J* = 8.1 Hz, 2H), 7.20–7.31 (m, 3H), 7.43 (d, *J* = 8.3 Hz, 2H); HRMS (ESI) calculated for C_42_H_57_N_12_O_8_ [M+H]^+^ 857.44168 found 857.44159 (Δ = –0.1 ppm)

1.3. Bioconjugation of Alkyne-Modified Nanoparticles with cRGD-Azide Derivative 3

The cRGD-azide derivative 3 (5 mg, 0.005 mmol) was added to a previously prepared suspension of alkyne-functionalized fibroin nanoparticles (20 mg, Lys: 80nmol/mg) in phosphate buffer (1 mL, pH 7.4). Afterwards, a 12 mg/mL solution of CuSO_4_ (100 μL, 0.0000481 mmol) and 52 mg/mL solution of sodium l-ascorbate (100 μL, 0.00262 mmol) were added. After 72 h, the *c*RGD-nanoparticles were purified through dialysis.

**Table S1.** SEC-UV analysis.

| **Protein** | **t_R_ ± SD (min)** | **V_elution_ ± SD (mL)** | **MW (kDa)** | **LogMW** |
| --- | --- | --- | --- | --- |
| Thyroglobulin | 5.898 ± 0.001 | 2.064 ± 0.000 | 660 | 1.137 |
| Rituximab | 7.903 ± 0.001 | 2.766 ± 0.000 | 144 | 1.563 |
| Bovine serum albumin | 8.755 ± 0.007 | 3.064 ± 0.002 | 67 | 1.823 |
| β-lactoglobulin | 9.509 ± 0.001 | 3.328 ± 0.000 | 36.8* | 2.158 |
| Ribonuclease A | 10.511 ± 0.003 | 3.679 ± 0.001 | 13.7 | 2.820 |

Data obtained from the SEC-UV analysis of five protein standards. Column: TSKgel SuperSW3000; mobile phase: 0.1 M Na2SO4, 0.05% (*w/v*) NaN3 in 0.1M phosphate buffer; pH 6.7. For further details, see the experimental section of the manuscript. SD = standard deviation. *molecular weight is 18400 Da, but at neutral pH, β-lactoglobulin exists in solution as dimeric form.

**Figure S1.** Calibration curve derived from the analysis in SEC-UV of five protein standards. Column: TSKgel SuperSW3000; mobile phase: 0.1 M Na_2_SO_4_, 0.05% (w/v) NaN_3_ in 0.1M phosphate buffer; pH 6.7. For further details, see the experimental section of the manuscript.

Text S2. Drug Release Studies

In vitro cumulative release of curcumin was investigated by the dialysis technique for both naked and functionalized nanoparticles. Briefly, for each sample, 15 mg of nanoparticles were suspended in 4 mL of deionized water and put into a dialysis membrane (3 – 5 kDa MWCO, Spectrum Laboratories, Milan, Italy). Each dialysis tube was incubated in 10 mL of ethanol/water 50% *v/v* and maintained under mild magnetic stirring at 37 °C. At each considered time point, all release medium was removed and replaced with fresh medium to ensure sink conditions. The amount of released drug was determined via a spectroscopic method (by reading the release media at 425 nm). A calibration curve was prepared (0.25–10.00 μg/mL, R^2^ = 0.989). For each formulation, three batches were analyzed. Raw data were processed using STATGRAPHICS XVII (Statpoint Technologies, Inc., Warrenton, Virginia, USA), and a linear generalized Analysis of Variance model (ANOVA) was used to evaluate the data. The post-hoc least significant difference (LSD) test for multiple comparisons was employed to analyze the differences between groups. The statistical significance was set up at *p* < 0.05. Data are reported as mean values and LSD intervals (*n* = 3).

.

**Figure S2.** In vitro drug release profiles of naked and functionalized SFNs. Data are reported as cumulative drug release percentage of at least three independent experiments. Data are reported as mean values ± LSD intervals, ANOVA. The overlap of two LSD intervals graphically indicates the absence of significant differences (*p* > 0.05).

**Table S2 (available in separate excel file).** Values of fluorescence intensity.

**Publisher’s Note:** MDPI stays neutral with regard to jurisdictional claims in published maps and institutional affiliations.

| 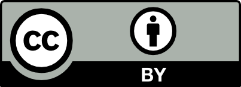 | © 2021 by the authors. Licensee MDPI, Basel, Switzerland. This article is an open access article distributed under the terms and conditions of the Creative Commons Attribution (CC BY) license (http://creativecommons.org/licenses/by/4.0/). |
| --- | --- |
